# Breastfeeding among parous women offered home-visit by a midwife after early discharge following planned cesarean section: Secondary analysis of a randomized controlled trial

**DOI:** 10.18332/ejm/173089

**Published:** 2023-12-07

**Authors:** Anne R. Kruse, Finn F. Lauszus, Axel Forman, Ulrik S. Kesmodel, Marie B. Rugaard, Randi K. Knud-en, Eva-Kristina Persson, Iben B. Sundtoft, Niels Uldbjerg

**Affiliations:** 1 Department of Obstetrics and Gynecology, Regional Hospital Godstrup, Herning, Denmark; 2 Department of Obstetrics and Gynecology, Aarhus University Hospital, Aarhus, Denmark; 3 Department of Clinical Medicine, Aarhus University, Aarhus, Denmark; 4 Department of Obstetrics and Gynecology, Aalborg University Hospital, Aalborg, Denmark; 5 Department of Clinical Medicine, Aalborg University, Aalborg, Denmark; 6 Department of Obstetrics and Gynecology, Regional Hospital Horsens, Horsens, Denmark; 7 Department of Health Sciences, Lund University, Lund, Sweden

**Keywords:** cesarean section, breastfeeding, self-efficacy, length of stay, postnatal care, house calls

## Abstract

**INTRODUCTION:**

Early discharge holds several advantages and seems safe after planned cesarean section among low-risk women. However, breastfeeding rates are lower after cesarean section. Thus, concern has been raised that early discharge among these women may affect breastfeeding even further. Therefore, we aimed to assess the effect of early discharge the day after planned cesarean section on breastfeeding, among parous women when a home-visit by a midwife was provided the day after discharge.

**METHODS:**

We conducted a secondary analysis of a randomized trial. Parous women (n=143) planned for cesarean section were allocated to either discharge within 28 hours after planned cesarean section followed by a home visit the day after (early discharge) or discharge at least 48 hours after planned cesarean section (standard care). The participants filled in questionnaires approximately 2 weeks before delivery and 1 week, 4 weeks, and 6 months postpartum.

**RESULTS:**

The proportions of women initiating breastfeeding were 84% versus 87% (early discharge vs standard care). After 6 months, 23% versus 21% were exclusively breastfeeding, while 29% versus 42% were partially breastfeeding. The mean duration of exclusive breastfeeding was 3.4 months (SD=2.3) in both groups. None of these differences was statistically significant. In both groups, the women’s breastfeeding self-efficacy score before cesarean section correlated with the duration of breastfeeding. After 4 weeks, low-score rates were 28% versus 30%.

**CONCLUSIONS:**

Early discharge with follow-up home visits by a midwife after planned cesarean section in parous women is feasible without compromising breastfeeding.

## INTRODUCTION

Early skin-to-skin contact and rooming-in after delivery are well-known factors in facilitating breastfeeding^1^. Hence, the first hours and days postpartum constitute a crucial period in enabling breastfeeding^1^. Even though WHO recommends exclusive breastfeeding until the child is 6 months old, studies report considerable variation in exclusive breastfeeding rates, e.g. 1–37% at 6 months postpartum^2-5^. Besides these differences between populations, the duration of breastfeeding is associated with several individual factors such as maternal education level, previous breastfeeding experiences, maternal breastfeeding self-efficacy score, and delivery mode^6-9^. Hence, compared to the situation after vaginal birth, breastfeeding may be even more challenging after a cesarean section (CS)^2,9-12^. Yet, once breastfeeding is established, the mode of delivery might not have an effect on breastfeeding continuation^8,9^. Therefore, the early postpartum period after CS seems to be of major importance.

During the last decades, early discharge after uncomplicated vaginal delivery has become more common^13^. This routine might increase the risk of breastfeeding problems, jaundice and re-admission^14,15^. However, it may hold several advantages for the family such as staying at home in a familiar environment, being with the entire family, and supporting the parental empowerment^13,16^. Also, after CS early discharge is practiced. Among low-risk women, this procedure is safe and feasible concerning postoperative recovery^17,18^. However, it remains unclear whether breastfeeding is affected by this routine. One may fear that the reduced time for inpatient guidance on breastfeeding compromises the initiation of breastfeeding. Published results on the topic are conflicting, i.e. an observational study demonstrated no association, while others report that late discharge had a positive effect on duration of exclusive breastfeeding^19-21^. However, the interpretation of these results is challenged by the designs of the studies, which hardly addressed contemporary definitions of early discharge and provided limited information on follow-up after discharge.

Offering postpartum home visits may promote early discharge and, further, support the sense of continuation and need of consistent advice requested by the women^3,22,23^. These home visits should include breastfeeding counselling, since breastfeeding support from a healthcare professional was found important for breastfeeding success^5,24,25^. When evaluating breastfeeding support from healthcare professionals, women reported positive factors such as an authentic presence, an empathetic approach, and an encouraging dialogue^26^. These elements might be easier to obtain during a home visit in which the woman feels comfortable and the interruptions are less, compared to during a hospital stay. Further, the provided home care may be more family centered and the partner is more likely to play a central supportive role also regarding breastfeeding in this setting^27^.

Previous randomized controlled trials (RCTs) on early discharge and enhanced recovery programs after CS have addressed breastfeeding as a secondary outcome^14,28-30^. A study from the United States showed that an enhanced recovery program after CS did not significantly increase the rate of discharge on day 2 (9% vs 3%), instead it increased exclusive breastfeeding at discharge (67% vs 48%) and continued breastfeeding 6 weeks postpartum (71% vs 38%)^29^. A Malaysian RCT found similar rate of exclusive breastfeeding 6 weeks postpartum when discharge was the first post-operative day compared to discharge on day 2 (45% vs 45%)^30^. On the other hand, an Egyptian RCT found that initiation of breastfeeding was negatively affected by discharge within 24 hours compared to discharge after 72 hours (62% vs 68%)^14^. Therefore, it remains unclear how breastfeeding is affected by the length of hospital stay, and especially how postpartum follow-up such as home-visits may alter this effect.

Besides measuring the proportion of women initiating breastfeeding and its duration, breastfeeding self-efficacy is also an outcome of interest. Self-efficacy in general is described by Bandura^31^ as a person’s belief in itself being able to exercise control and thereby manage a situation or task. It is determined by internal personal factors, but also by the external environment. The women’s attitude towards breastfeeding can be assessed by the breastfeeding self-efficacy score. Studies have found that breastfeeding self-efficacy score is correlated to duration of breastfeeding^32^.

Even though early discharge seems safe after planned CS among low-risk women^33,34^, the studies mentioned above allow no definite conclusions on the effect on breastfeeding. Therefore, we performed a secondary analysis of a Danish RCT on early discharge after planned CS (≤28 vs >48 hours). The secondary outcomes were initiation of breastfeeding, self-efficacy score, and duration of breastfeeding.

## METHODS

### Setting

This study included parous women planned for term CS at two obstetrical departments in Denmark from September 2016 to September 2019. The method was previously described in detail^18^. The primary outcome of the RCT was parental sense of security compared between two groups allocated for either standard or early discharge. The latter was offered a home visit by a midwife. In this study, we performed a secondary analysis of breastfeeding in this setting.

### Inclusion and exclusion criteria

The inclusion criterion was planned CS in a parous woman. Exclusion criteria were multiple pregnancy, pre-pregnancy BMI ≥35 kg/m^2^, age <18 years, inability to read and write Danish, living alone, planned prolonged observation of the woman or the new-born, and previous negative breastfeeding experiences, leading to a planned prolonged hospital stay.

### Participants

The participants were recruited from the outpatient clinic when the planned CS was decided. They received written information and oral information by telephone. After informed consent was obtained, the participants were randomly allocated 1:1 to either early discharge or standard care. This allocation took place approximately 2 weeks before the planned CS.

*Intervention (early discharge group)*

Discharge was intended within 28 hours after CS. These women were offered a home visit by a midwife the day after discharge. The home visit included guidance on breastfeeding in addition to standard examinations of the newborn, weight control, a hearing test, and dried blood spot screening for congenital conditions.

*Control (standard care group)*

Discharge was intended at least 48 hours after CS. Standard examinations of the newborn, weight control, a hearing test, and dried blood spot screening for congenital conditions were performed before discharge. Women in this group were offered guidance on breastfeeding during hospital stay but were not offered a home visit.

Otherwise, both groups received the same standard perioperative care during hospital stay and fulfilled the same criteria for discharge^18^. Decision on whether the woman was ready for discharge as intended was made between the woman and the personnel. Both groups received the same standard home visits by a local child healthcare nurse about 4 to 5 days after birth.

### Outcomes

The outcomes were initiation and duration of breastfeeding and breastfeeding self-efficacy score. Breastfeeding self-efficacy score^31,32^ was measured using the question: ‘How certain are you that you will breastfeed exclusively until 4 months postpartum?’. The response options were: ‘very certain’, ‘certain’, ‘do not know’, ‘uncertain’, and ‘very uncertain’. This question was answered in a diary approximately 2 weeks before planned birth and at 1 week and 4 weeks postpartum. Uncertain and very uncertain were categorized as low breastfeeding self-efficacy.

Six months postpartum, participants received a questionnaire by e-mail regarding duration of exclusive and partial breastfeeding. Breastfeeding was defined as partial if the child received anything else than mother’s milk, such as formula or any kind of complementary food. Duration was reported in numbers of months, rounded up to the nearest half month.

### Statistical analysis

Sample size was based on the primary outcome of the RCT^18^. Data were analyzed using STATA 17 (College Stations, Texas, USA). Analyses were performed according to the intention-to-treat principle. Chi-squared or Fisher’s exact test, as appropriate, were used for categorized outcomes. Breastfeeding self-efficacy and duration of breastfeeding were analyzed using linear regression and survival analysis. A two-sided p<0.05 was chosen as level of significance.

## RESULTS

A total of 266 parous women fulfilled the inclusion criteria. Of these, 143 parous women (54%) accepted participation. Thus, 72 were allocated to early discharge (≤28 hours after CS) and 71 to standard care (discharge >48 hours after CS). The women in the two groups did not differ regarding their basic characteristics (Table 1)^18^. Before the CS, 89% in the early discharge group and 90% in the standard care group planned to breastfeed.

**Table 1:** Characteristics of the women included in the randomized controlled trial scheduled for planned cesarean section, Denmark, 2016–2019 (N=143)

*Characteristics*	*Early discharge group (N=72)*	*Standard care group (N=71)*
**Maternal age** (years), mean (SD)	33.4 (4.5)	32.5 (4.6)
**BMI** (kg/m^2^), mean (SD)	25.8 (4.0)	25.6 (4.3)
**Smoking during pregnancy**, % (n/N)	11 (8/72)	6 (4/71)
**Education level^a,b^**, % (n/N) Low Medium High	9 (5/54) 33 (18/54) 57 (31/54)	6 (3/52) 21 (11/52) 73 (38/52)
**Length of hospital stay** (hours), median (IQR),	27.4 (26.0–34.5)	50.9 (49.6–52.7)
**Planned to breastfeed^b^,** % (n/N) Yes No Do not know	89 (48/54) 7 (4/54) 4 (2/54)	90 (47/52) 4 (2/52) 6 (3/52)

a Low: Primary or upper secondary education. Medium: vocational education and training and qualifying educational programs, short cycle higher education and vocational/Bachelor’s education. High: medium cycle higher education/Bachelor’s programs, long cycle higher education/Master’s or PhD programs.

b Among 54 women answering in the early discharge group and 52 women answering in the standard care group. IQR: interquartile range. p=0.24 (Fisher’s exact test).

**Figure 1:**
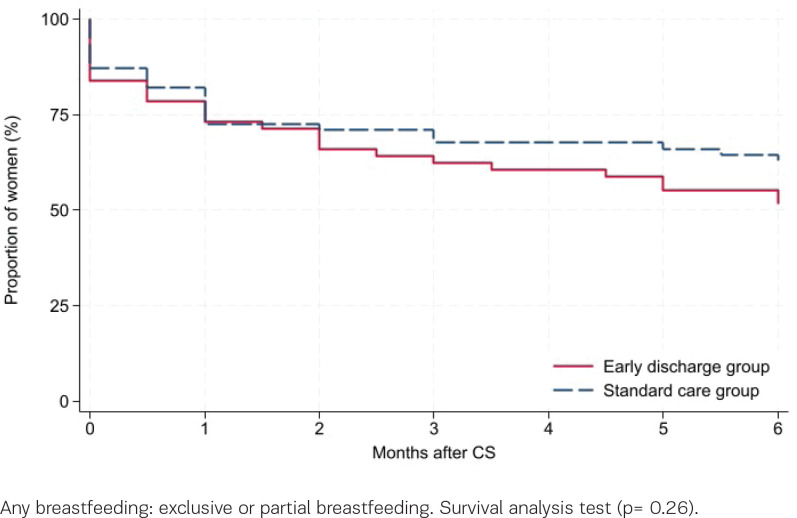
Any breastfeeding after planned cesarean section among women randomized for early discharge or standard care, Denmark, 2016–2019 (N=118)

**Figure 2:**
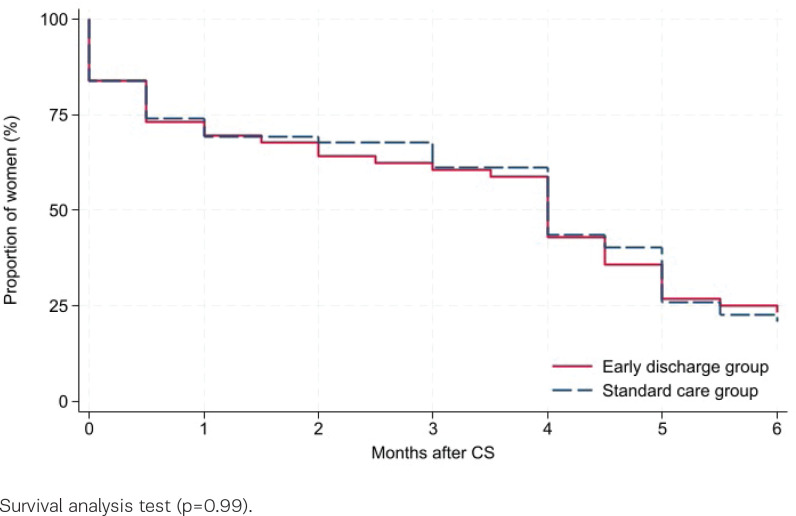
Exclusive breastfeeding after planned cesarean section among women randomized for early discharge or standard care, Denmark, 2016–2019 (N=118)

Of the 143 participants, 75% (54/72) and 73% (52/71), early discharge and standard care, respectively, filled in the diary within the first month after CS. Six months postpartum, 78% (56/72) and 87% (62/71) answered the follow-up questionnaire. Among the responders, 84% versus 87% initiated breastfeeding (early discharge vs standard care, p=0.63, Table 2). Six months postpartum, 23% versus 21% were exclusively breastfeeding (p=0.77), while 29% versus 42% were partially breastfeeding (p=0.13). The mean duration of exclusive breastfeeding was 3.4 months (SD=2.3) in both groups (p=0.85). Survival analysis did not reveal any differences in duration of breastfeeding between the groups, neither regarding any breastfeeding (p=0.26, Figure 1) nor exclusive breastfeeding (p=0.99, Figure 2). A subgroup analysis comparing women discharged ≤28 versus >48 hours after CS, regardless of group allocation, found comparable results (p=0.98 and 0.49, respectively, Supplementary file Figure 1).

**Table 2:** Breastfeeding among women in early discharge versus standard care group, answering a questionnaire 6 months after planned cesarean section, in a randomized controlled trial, Denmark, 2016–2019 (N=118)

	*Early discharge group (N=56)*		*Standard care group (N=62)*		*p^a^*
	*n*	*% (95% CI)*	*n*	*% (95% CI)*	
**Breastfeeding initiated**	47	84 (72–92)	54	87 (76–94)	0.63
**Breastfeeding at 6 months** Total	29	52 (38–65)	39	63 (50–75)	0.22
Exclusive	13	23 (13–36)	13	21 (12–33)	0.77
Partial	16	29 (17–42)	26	42 (30–55)	0.13

a Chi-squared test.

We found a strong association between maternal breastfeeding self-efficacy and duration of breastfeeding (p<0.001, linear regression, Table 3) with no significant differences between the groups (Table 4). Low breastfeeding self-efficacy score was reported among 19% versus 24% before and 28% versus 30% at 4 weeks after CS (Table 4).

**Table 3:** Breastfeeding self-efficacy before cesarean section and duration of breastfeeding among women answering the questionnaire before and after cesarean section, in a randomized controlled trial, Denmark, 2016–2019 (N=91)

*Breastfeeding self-efficacy^a^ (score)*	*n*	*Breastfeeding duration (months) Mean (95% CI)*
Very certain (1)	36	5.0 (4.4–5.5)
Certain (2)	25	4.3 (3.6–5.0)
Do not know (3)	14	2.7 (1.8–3.6)
Uncertain (4)	9	2.2 (1.0–3.3)
Very uncertain (5)	7	1.1 (-0.12–2.4)

a ‘How certain are you that you will breastfeed exclusively until 4 months postpartum?’. p<0.001, linear regression.

**Table 4:** Proportion of women undergoing planned cesarean section with low breastfeeding self-efficacy score^a^, in a randomized controlled trial, Denmark, 2016–2019

	*Early discharge group*		*Standard care group*		*p^b^*
	*n/N*	*% (95% CI)*	*n/N*	*% (95% CI)*	
Before CS	10/52	19 (10–33)	12/51	24 (13–38)	0.60
1 week after CS	11/49	22 (12–37)	12/44	27 (15–43)	0.59
4 weeks after CS	14/50	28 (16–43)	14/47	30 (17–45)	0.85

a Low breastfeeding self-efficacy: score 4 (uncertain) and 5 (very uncertain).

b Chi-squared test.

## DISCUSSION

In this study, we randomized parous women undergoing planned CS to either early discharge (≤28 hours after CS) followed by a home visit or standard care (discharge >48 hours after CS) and found no significant differences in breastfeeding initiation, duration, or the rate of women with low breastfeeding self-efficacy score.

Previous studies also found that breastfeeding self-efficacy, as well as breastfeeding in general, is impaired among women after CS^10,11^. This may indicate that these women need intensive breastfeeding support under as optimal conditions as possible. In our study population, we found a total of 21% (19% vs 24%, respectively, in the two groups) with low breastfeeding self-efficacy before CS. This is comparable to the 34% found in another Danish study, which in contrast to our study included women regardless of parity and delivery mode^32^. Since the breastfeeding self-efficacy score predicts increased risk of early breastfeeding cessation, this may help us identify women appropriate for increased support either during hospital stay or in their home surroundings.

Three other RCTs^14,29,30^ support our results regarding breastfeeding after early discharge, including post-discharge follow-up after planned CS in parous women. One of these RCTs was conducted in Malaysia, where the University hospital ‘offered comprehensive healthcare to the public at subsidized rates or for free’^30^. The study included 360 mostly parous women undergoing planned CS allocated to intended discharge on day 1 or day 2. After discharge, the women had free access to the clinic, which was open at all hours, whereas they did not receive home visits. At 6 weeks, the rate of exclusive breastfeeding was 45% in both groups. Another RCT was conducted in Egypt, where the University Clinic in Cairo offered ‘medical service totally free of any charges’ including 2998 mostly parous women allocated to discharge after 24 or 72 hours following planned or acute CS^14^. After discharge the women had access to medical help in an outpatient clinic. The rates of breastfeeding after 6 weeks were 62% and 68%, respectively. Despite these relatively high breastfeeding rates, they found a significant difference between the groups (p=0.001). Yet, one may speculate if postpartum home visits would have levelled out this difference.

A limitation of RCTs on topics like breastfeeding is that they do not account for the birth and the establishment of parenthood as a life transition, which deserves individualized managing^35^. Thus, a meta-synthesis on parental experiences of early postnatal discharge concluded that ‘the mothers’ and fathers’ experiences of responsibility, security and confidence in their parental role, were positively influenced by having the opportunity to be together as a family, receiving postnatal care that included both parents, having influence on time of discharge, and getting individualized and available support focused on developing and recognizing their own experiences of taking care of the baby’^16^. This emphasizes the importance of individualized and modifiable plans for length of hospital stay as well as for the guidance regarding breastfeeding^3,16,26,36^. Therefore, it is essential that we do not implement early discharge at the expense of shared decision-making^16^.

Even though we did not find a significant difference in breastfeeding rates when comparing women discharged on day 1 and 2, it is important to acknowledge that women often report breastfeeding difficulties on day 3, which increases the risk of cessation. Therefore, optimal organization, extent, and timing of follow-up after discharge constitute important future research topics; not only for women delivered by planned CS but for all women who intend to breastfeed.

### Strengths and limitations

The strengths of the study include the randomized design and the high response rate (83%) to the questionnaires. It is a limitation that it is a secondary analysis; thus, the sample size was not based on breastfeeding outcomes (i.e. the results are only hypothesis generating). Further, only 54% of eligible patients accepted participation, which may have induced recruitment bias and, hence, affected the internal validity. Also, the external validity may be compromised by the Danish setting. Danish culture supports a strong tradition for breastfeeding, including focus on skin-to-skin contact supported by the staff within the first hours after birth. Furthermore, the Danish healthcare system already offers a postpartum follow-up provided by a local child healthcare nurse starting within the first week after birth.

## CONCLUSIONS

Among low-risk parous women undergoing planned CS, early discharge after shared decision-making does not compromise breastfeeding. However, a prerequisite for this conclusion might be the inclusion of home visits by a healthcare professional with expertise in breastfeeding. Regarding the importance of individualized plans for the women, further research is needed.

## Data Availability

The data supporting this research cannot be made available for privacy or other reasons. Supply of data to other parties was not included in the approval from the Danish Data Protection Agency or the Central Denmark Region Ethics Committee.
