# Supplementary material for: Breastfeeding among parous women offered home-visit by a midwife after early discharge following planned cesarean section: Secondary analysis of a randomized controlled trial
Source: Eur J Midwifery. 2023 Dec 7;7:38. doi: 10.18332/ejm/173089 (PMC10701761; doi:10.18332/ejm/173089)
Supplement: Supplementary file 1 [file EJM-7-38-s1.pdf]

Supplementary figure 1

Intention-to-treat analysis
(Figure 1 & 2)

Any breastfeeding (p=0.2567):

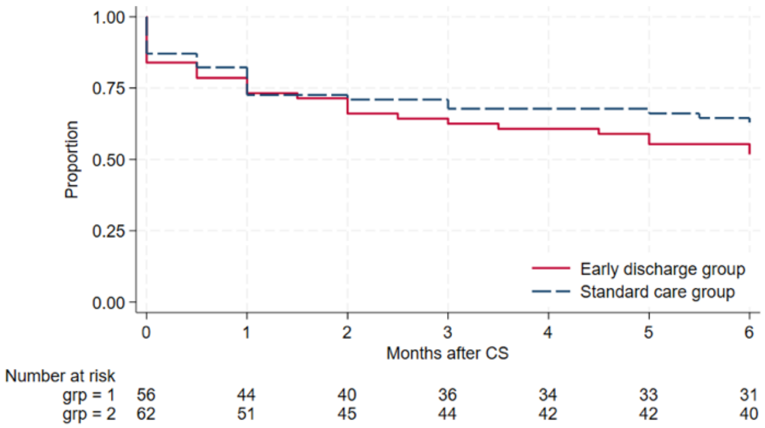

Exclusive breastfeeding (p=0.9914):

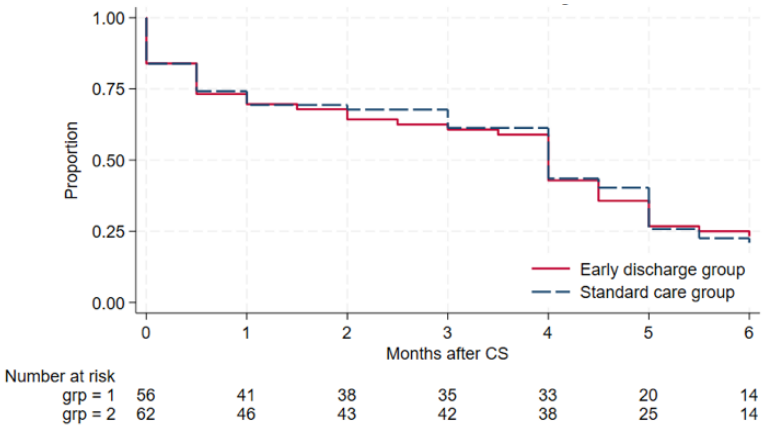

Subgroup analysis
Breastfeeding among 41 women discharged within 28 hours and 71 women discharged after 48 hours after planned cesarean section

Any breastfeeding (p=0.9773):

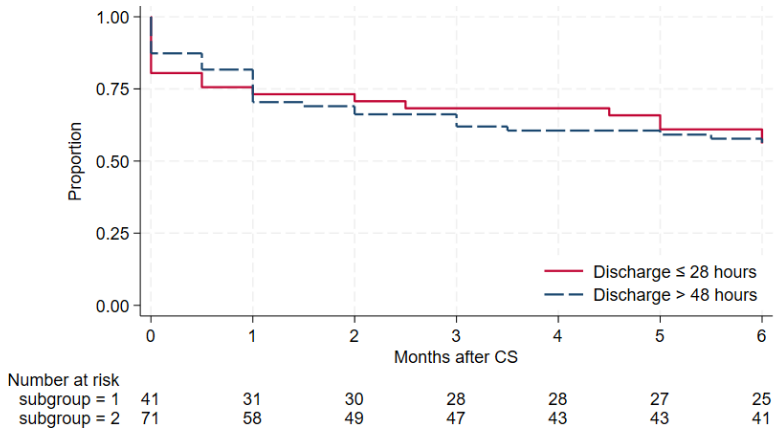

Exclusive breastfeeding (p=0.4865):

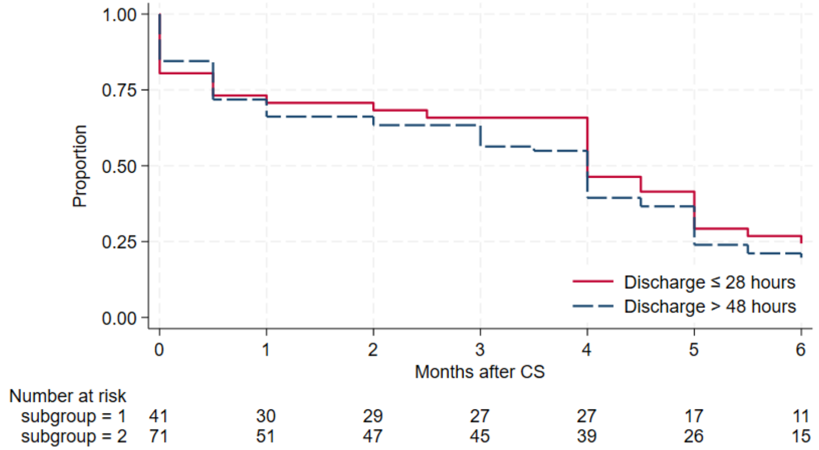

© 2023 Kruse A. R. et al.
